# Application of the 21-Gene Recurrence Score in Patients with Early HR-Positive/HER2-Negative Breast Cancer: Chemotherapy and Survival Rate According to Clinical Risk

**DOI:** 10.3390/cancers13164003

**Published:** 2021-08-09

**Authors:** Soong June Bae, Sung Gwe Ahn, Jung Hwan Ji, Chihhao Chu, Dooreh Kim, Janghee Lee, Yoon Jin Cha, Joon Jeong

**Affiliations:** 1 Department of Surgery, Gangnam Severance Hospital, Yonsei University College of Medicine, 211 Eonju-ro, Gangnam-gu, Seoul 06273, Korea; mission815815@yuhs.ac (S.J.B.); ASG2004@yuhs.ac (S.G.A.); SHEVCHENCKO@yuhs.ac (J.H.J.); WONYHAPPY@yuhs.ac (C.C.); 2 Institute for Breast Cancer Precision Medicine, Yonsei University College of Medicine, 237 Dogok-ro, Gangnam-gu, Seoul 06230, Korea; YOONCHA@yuhs.ac; 3 Department of Surgery, Seoul St. Mary’s Hospital, College of Medicine, The Catholic University of Seoul, Seoul 06591, Korea; rlaenfpd@naver.com; 4 Department of Surgery, Sacred Heart Hospital, Hallym University, Hwaseong 18450, Korea; Doctorlee85@outlook.kr; 5 Department of Pathology, Gangnam Severance Hospital, Yonsei University College of Medicine, 211 Eonju-ro, Gangnam-gu, Seoul 06273, Korea

**Keywords:** breast neoplasms, adjuvant treatment, 21-gene recurrence score, chemotherapy, clinical risk

## Abstract

**Simple Summary:**

It is important to address the influence of 21-gene Recurrence Score (RS) on chemotherapy decision-making stratified by clinical risk in patients with hormone receptor (HR)-positive/human epidermal growth factor receptor 2 (HER2)-negative early breast cancer. Our study presented that the application of the 21-gene RS assay significantly reduced the chemotherapy rate in patients with high clinical risk. Meanwhile, there was no significant difference in the chemotherapy rate according to the implementation of the 21-gene RS assay in those with low clinical risk. Furthermore, we observed no difference in prognosis according to the application of 21-gene RS for either clinical risk. These results suggest that the 21-gene RS could be considered more positively in HR+/HER2- patients with high clinical risk to reduce chemotherapy rates without increasing the occurrence of relapse.

**Abstract:**

We assessed the impact of 21-gene Recurrence Score (RS) assay on chemotherapy decision-making according to binary clinical risk stratification in patients with hormone receptor (HR)-positive/human epidermal growth factor receptor 2 (HER2)-negative early breast cancer. We included patients with tumors measuring 1–5 cm, N0-1, and HR+/HER2- breast cancer who underwent surgery followed by adjuvant treatment. The clinical risk was determined by a modified version of Adjuvant! Online. We performed propensity score matching (PSM) according to the application of 21-gene RS separately in the low and high clinical risk groups. Before PSM, 342 (39.0%) of 878 patients were classified as having high clinical risk. In the high clinical risk group, 21-gene RS showed a significantly reduced chemotherapy rate of 39.3%, without increasing the recurrence. After PSM, the 21-gene RS application significantly reduced chemotherapy rate by 34.0% in 200 patients with high clinical risk (21-gene RS application, 32.0% vs. no 21-gene RS application, 66.0%, *p* < 0.001). There was also no significant difference in RFS according to 21-gene RS status in the high clinical risk group (log-rank test, *p* = 0.467). These results support the usefulness of the 21-gene RS to reduce the chemotherapy rate without adversely affecting prognosis in a high clinical risk group.

## 1. Introduction

The 21-gene recurrence score (RS) assay (Oncotype DX, Genomic Heal, Redwood City, CA, USA) is one of the most frequently used commercially available gene-expression assays in breast cancer [1,2]. The 21-gene RS based on Oncotype DX assay was initially developed to quantify the likelihood of distant recurrence in women with hormone receptor (HR)-positive/human epidermal growth factor receptor 2 (HER2)-negative, node-negative breast cancer, with a high RS on a scale of 0 to 100 indicating a higher risk of distant recurrence [3,4]. Although the adjuvant chemotherapy reduced the risk of distant recurrence [5,6,7], there is a concern that the chemotherapy is unnecessary in the majority of patients with HR-positive, HER2-negative breast cancer. The predictive value of the 21-gene RS for chemotherapy benefit in women with ER-positive, HER2-negative breast cancer has been validated in several prospective clinical trials, including the National Surgical Adjuvant Breast and Bowel Project B-20 trial, Southwest Oncology Group (SWOG)-8814 trial, and Trial Assigning Individualized Options for Treatment (TAILORx) [8,9,10,11]. The chemotherapy benefit was observed when the 21-gene RS was high, whether a high 21-gene RS was defined as 31 or higher, or 26 or higher. Based on these results, the National Comprehensive Cancer Network guidelines recommend strong consideration of the 21-gene RS assay to determine the adjuvant chemotherapy in patients with tumor size > 0.5 cm, N0, and HR-positive/HER2-negative breast cancer [12]. Consequently, the use of the 21-gene RS has led to a decline in the chemotherapy rate sparing serious toxicities for HR-positive, HER-negative breast cancer in clinical practice [13,14,15,16].

The TAILORx trial was designed to address whether the adjuvant chemotherapy is beneficial for patients with HR+/HER2-, node-negative breast cancer with a midrange RS of 11 to 25, and revealed that adjuvant endocrine therapy alone was not inferior compared to adjuvant chemotherapy plus endocrine therapy [6]. Moreover, the results of a secondary analysis of the TAILORx suggested that the integration of the 21-gene RS and clinical risk provided more accurate information on the prognosis and the chemotherapy benefit of individual patients [17]. However, whether the application of 21-gene RS has an advantage in terms of reduction for adjuvant chemotherapy without adverse survival outcomes stratified by clinical risk has not yet been reported.

This study investigated the chemotherapy rates in the patient groups stratified by clinical risk according to 21-gene RS use. We also analyzed the impact of 21-gene RS on survival outcomes according to clinical risk.

## 2. Materials and Methods

### 2.1. Study Population

Following the Good Clinical Practice guidelines and the principles of the Declaration of Helsinki, our study was approved by the Institutional Review Board at Gangnam Severance Hospital, Yonsei University, Seoul, Republic of Korea (IRB no. 3-2021-0042), which waived the requirement for informed consent due to the retrospective study design.

**Figure 1:**
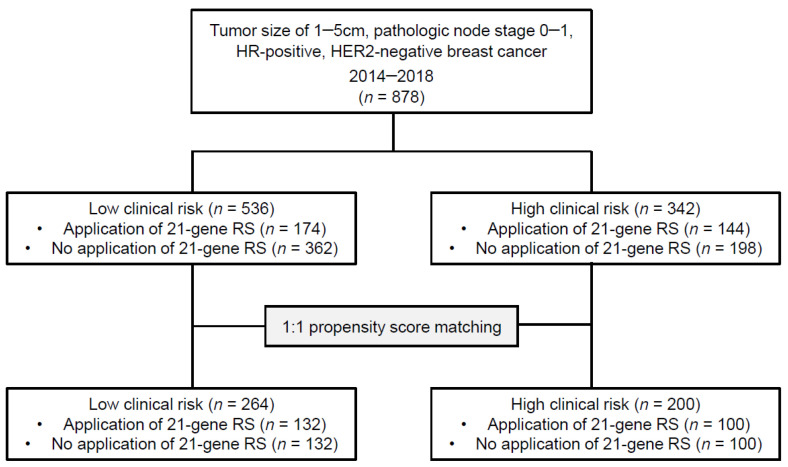
Study flowchart.

The medical records of patients with breast cancer who underwent breast surgery followed by adjuvant treatment at Gangnam Severance Hospital between January 2014 and December 2018 were reviewed. We identified 878 patients with tumor size of 1–5 cm, pathologic node stage 0–1, HR-positive, HER2-negative breast cancer (Figure 1). The available clinicopathologic data included age, type of surgery, adjuvant treatment including chemotherapy and endocrine therapy, survival, ER status, progesterone receptor (PR) status, HER2 status, Ki-67 levels, histologic type, histologic grade (HG), lymphovascular invasion (LVI) status, pathologic stage, and 21-gene RS. The clinical risk was assessed using the Adjuvant! Algorithm as described in the MINDACT trial [18,19]. We classified the clinical risk as low or high based on the tumor size, nodal metastasis, and HG. Briefly, in N0 patients, the clinical risk was defined as low if the tumor was ≤3 cm in diameter and had a low HG, ≤2 cm in diameter and had an intermediate HG, or ≤1 cm in diameter and had a high HG. In N1 patients, the clinical risk was defined as low if the tumor was ≤2 cm in diameter and had a low HG. The clinical risk was defined as high if the low-risk criteria were not met.

Oncotype DX 21-gene RS assays were not routinely performed in our institution because they are not covered by insurance. The clinicians discussed the use of the 21-gene RS with the patient based on the patient’s clinicopathologic risk factors as well as their personal preferences. Previous studies, which predicted 21-gene RS results with clinicopathologic data, reported that the age, tumor size, HG, PR, LVI, and Ki-67 were related to the RS [20,21,22]. Based on these, the Oncotype DX assay was frequently omitted in patients whose clinicopathologic features were either mostly favorable or poor because their 21-gene RS was strongly expected to be low or high. In contrast, the clinicians generally recommended the application of the 21-gene RS assay to patients with intermediate or mixed clinicopathologic data. Consequently, 318 (36.2%) patients underwent 21-gene RS testing.

### 2.2. Immunohistochemistry

In our immunohistochemistry study, formalin-fixed, paraffin-embedded tissue sections obtained from surgical specimens were stained using appropriate antibodies specific for four markers: ER (1:100 dilution, clone 6F11; Novocastra, Newcastle upon Tyne, UK), PR (clone 16; Novocastra, UK), HER2 (4B5 rabbit monoclonal antibody; Ventana Medical Systems, Tucson, AZ, USA), and Ki-67 (MIB-1; Dako, Glostrup, Denmark). According to the modified Allred system, ER and PR positivity were defined as Allred scores of 3–8, while negativity was defined as Allred scores of 0 and 2, respectively. We considered Allred scores of 7–8 to indicate high expression levels. HER2 status was defined as positive for scores of 3+ and negative for scores of 0 or 1+. Tumors with scores of 2+ were sent for fluorescent in situ hybridization analysis according to the suppliers’ protocols (PathVysion kit; Vysis, Downers Grove, IL, USA, or HER2 inform; Ventana). We defined Ki-67 levels ≥14% as high.

### 2.3. 21-Gene RS Assay

The 21-gene RS assay (Oncotype DX, Genomic Health, Redwood City, CA, USA) is based on reverse transcriptase-polymerase chain reaction (RT-PCR) that can be performed on the RNA isolated from formalin-fixed paraffin-embedded (FFPE) tissue [3]. After reviewing the hematoxylin and eosin-stained slides to determine whether sufficient invasive breast cancer was present and whether manual microdissection was indicated, RNA extraction from the unstained sections and the 21-gene RS assay was performed by Genomic Health (Redwood City, CA, USA). This assay evaluates the expression of 16 tumor-associated genes (ER, PGR, BCL2, SCUBE2, GRB7, HER2, Ki-67, STK15, Survivin, CCNB1, MYBL2, MMP11, CTSL2, GSTM1, CD68, BAG1) and 5 reference genes (ACTB, GAPDH, RPLPO, GUS, TFRC) by using RT-PCR. Based on the expression levels of 21-genes, an algorithm was designed to compute a 21-gene RS for each sample. Quantitative single-gene scores for ER and PR mRNA expression, determined via an RT-PCR, were also provided within the final assay report by Genomic Health.

### 2.4. Statistical Analysis

Categorical variables were compared by chi-square tests. The chemotherapy rates according to the clinical risk were analyzed by chi-square or Fisher’s exact tests. Binary logistic regression analysis was performed to identify independent factors associated with a reduced chemotherapy rate. Variables with *p* < 0.10 in univariable analysis were included in the multivariable analysis. We performed an individual propensity score matching (PSM) analysis in which randomly selected patients with 21-gene RS were paired with comparable patients without 21-gene RS. The one case per one control was selected based on age (≤50 vs. >50 years), histologic type (invasive ductal carcinoma vs. invasive lobular carcinoma vs. others), histologic grade (I or II vs. III), ER (Allred score 0–6 vs. 7–8), PR (Allred score 0–6 vs. 7–8), Ki-67 (<14% vs. ≥14%), pathologic T stage (1 vs. 2), and pathologic *n* stage (0 vs. 1). Recurrence-free survival (RFS) was measured as the period from the date of breast cancer surgery to first breast cancer recurrence, including loco-regional and distant recurrences. The Kaplan–Meier method with a log-rank test was used to calculate the RFS and compare the results between groups. All analyses were performed using IBM SPSS Statistics for Windows, version 23.0 (IBM Corp.; Armonk, NY, USA) and SAS (version 9.3, SAS Inc., Cary, NC, USA). The statistical significance was defined as *p* < 0.05.

## 3. Results

### 3.1. Baseline Characteristics

The median age of all patients was 52 years (range, 25–87). Among all patients, 536 (61.0%) and 342 (39.0%) were at low and high clinical risk, respectively (Figure 1). As expected, patients with high clinical risk had more poor prognostic factors such as high HG, Ki-67, pathologic T stage, pathologic *n* stage, LVI, and low PR expression compared to those in patients with low clinical risk. The chemotherapy rate (52.6% vs. 13.2%, *p* < 0.001), and implementation of 21-gene RS (42.1% vs. 32.5%, *p* = 0.004) were significantly higher in patients with high clinical risk. Moreover, patients with high clinical risk and 21-gene RS had favorable clinicopathologic factors, while the reverse trend was observed in patients with low clinical risk (Table S1). Meanwhile, the distributions of categorical 21-gene RS did not differ according to clinical risk (Figure S1); the results were consistent using different 21-gene RS cutoffs used in clinical trials; namely: low (<18 or <11), intermediate (18–30 or 11–25), and high (≥31 or >25).

To minimize the baseline confounders affecting the chemotherapy rate, a one-to-one PSM between patients with or without 21-gene RS was carried out separately in the low and high clinical risk groups. Of 464 patients (median age, 52 years; range, 25–87) in the PSM cohort, 264 (56.9%) showed low clinical risk, while 200 (43.1%) showed high clinical risk. After PSM, all variables were well balanced according to the implementation of 21-gene RS in both clinical risk groups (Table 1).

### 3.2. Chemotherapy Rates

**Figure 2:**
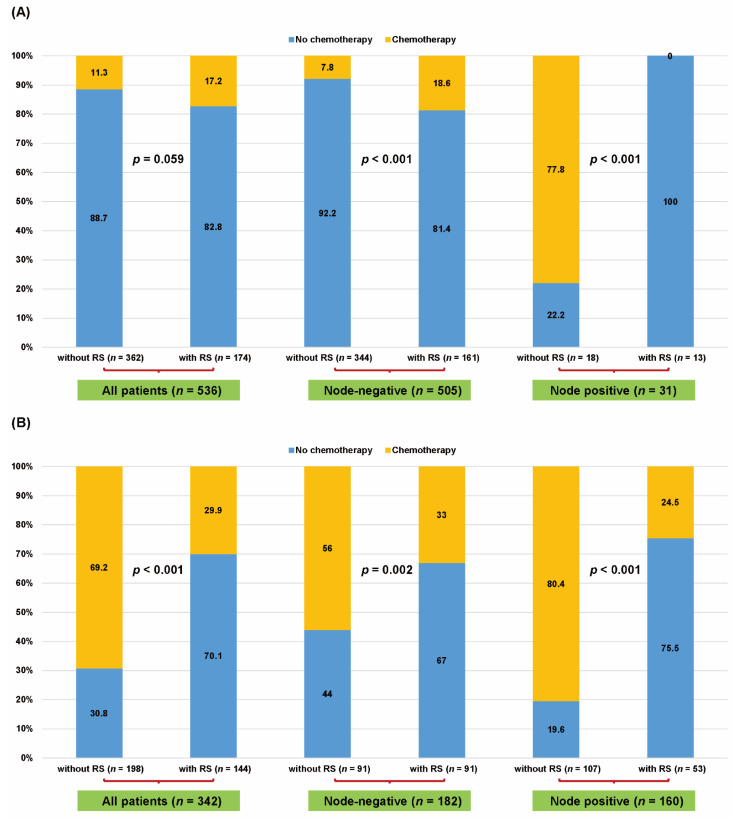
Chemotherapy rates according to the application of the 21-gene Recurrence Score (RS) assay in the entire cohort. Chemotherapy rates according to the application of the 21-gene RS assay stratified by clinical risk in patients with (**A**) low clinical risk and (**B**) high clinical risk. Chi-square test, significance level of 0.05.

Among the 536 patients with low clinical risk, 41 of 362 (11.3%) without 21-gene RS and 30 of 174 (17.2%) with 21-gene RS received chemotherapy (*p* = 0.059). Among patients with node-negative and low clinical risk, 27 of 344 (7.8%) without 21-gene RS and 30 of 161 (18.6%) with 21-gene RS received chemotherapy (*p* < 0.001). Among patients with node-positive and low clinical risk, 14 of 18 (77.8%) without 21-gene RS and none of the patients with 21-gene RS received chemotherapy (*p* < 0.001, Figure 2A). Meanwhile, among the 342 patients with high clinical risk, 137 of 198 (69.2%) without 21-gene RS and 43 of 144 (29.9%) with 21-gene RS received chemotherapy (*p* < 0.001). Among patients with the node-negative disease and high clinical risk, 51 of 91 (56.0%) without 21-gene RS and 30 of 91 (33.0%) with 21-gene RS received chemotherapy (*p* = 0.002). Among patients with node-positive disease and high clinical risk, 86 of 107 (80.4%) without 21-gene RS and 13 of 53 (24.5%) with 21-gene RS received chemotherapy (*p* < 0.001, Figure 2B). In the multivariable analysis, the implementation of the 21-gene RS was an independent factor associated with a reduced chemotherapy rate in the high clinical risk group (odds ratio (OR) 0.196; 95% confidence interval (CI), 0.115–0.334; *p* < 0.001, Table 2). Furthermore, the implementation of 21-gene RS was significantly associated with a reduced chemotherapy rate in both N0 (OR 0.357; 95% CI, 0.176–0.724; *p* = 0.004) and N1 (OR 0.085; 95% CIs, 0.036–0.200; *p* < 0.001) disease.

**Figure 3:**
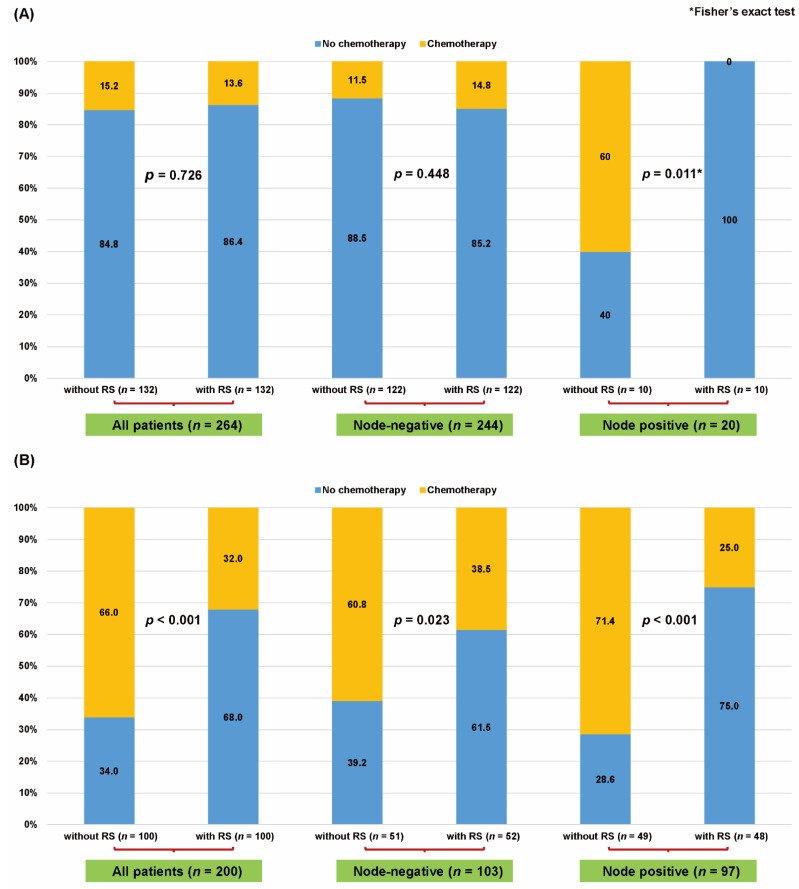
Chemotherapy rates according to the application of the 21-gene Recurrence Score (RS) assay in the propensity-Scheme 21-gene RS assay stratified by clinical risk in patients with (**A**) low clinical risk and (**B**) high clinical risk. Chi-square test, significance level 0.05. * Fisher’s exact test, significance level of 0.05.

After PSM, the chemotherapy rates did not differ significantly according to 21-gene RS status in women with low clinical risk (15.2% (20 of 132) in patients without 21-gene RS vs. 13.6% (18 of 132) in patients with 21-gene RS, *p* = 0.726) and those with low clinical risk and node-negative disease (11.5% (14 of 122)in patients without 21-gene RS vs. 14.8% (18 of 122) in patients with 21-gene RS, *p* = 0.448, Figure 3A). Otherwise, use of the 21-gene RS was associated with significantly reduced chemotherapy rates in women with low clinical risk and node-positive disease (60.0% (6 of 10)in patients without 21-gene RS vs. none in patients with 21-gene RS, *p* = 0.011). Likewise, use of the 21-gene RS was associated with a significantly decreased chemotherapy rate in women with high clinical risk (66.0% (66 of 100) in patients without 21-gene RS vs. 32.0% (32 of 100) in patients with 21-gene RS, *p* < 0.001), high clinical risk and node-negative disease (60.8% (31 of 51) in patients without 21-gene RS vs. 38.5% (20 of 52) in patients with 21-gene RS, *p* = 0.023), and high clinical risk and node-positive disease (71.4% (35 of 49) in patients without 21-gene RS vs. 25.0% (12 of 48) in patients with 21-gene RS, *p* < 0.001, Figure 3B).

### 3.3. Clinical Outcomes

**Figure 4:**
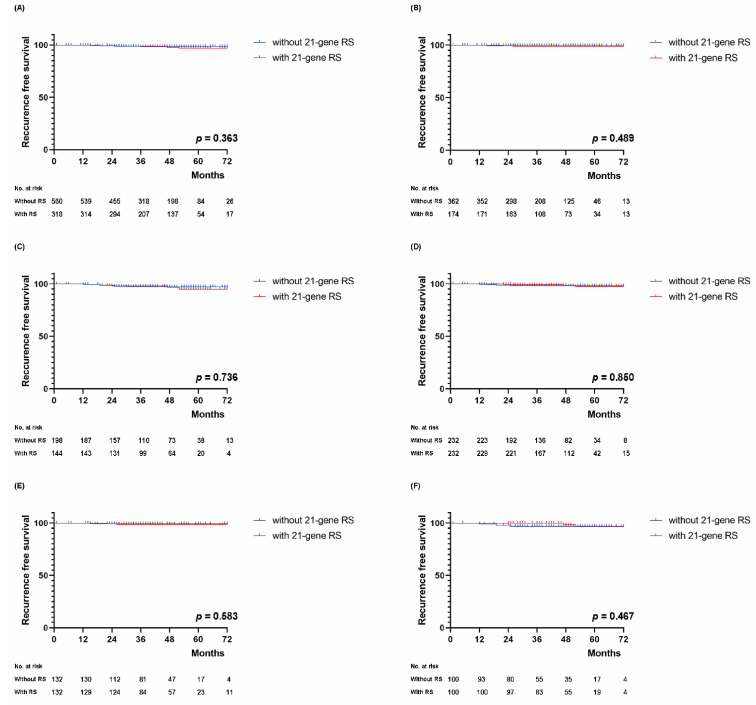
Recurrence-free survival (RFS) according to the application of the 21-gene Recurrence Score (RS) assay. Kaplan–Meier curves of RFS according to the application of the 21-gene RS (**A**) in all patients, (**B**) in all patients with low clinical risk, and (**C**) in all patients with high clinical risk, as well as (**D**) in the entire propensity-score matching (PSM) cohort, (**E**) in the PSM cohort with low clinical risk, and (**F**) in the PSM cohort with high clinical risk. Log-rank test, significance level of 0.05.

In the entire cohort, there were 14 recurrence events and no deaths during the median follow-up of 42 months (range, 1–83 months). There was no significant difference in RFS according to 21-gene RS status (log-rank test, *p* = 0.363; Figure 4A). In addition, RFS did not differ according to 21-gene RS status in both groups stratified by clinical risk (*p* = 0.489 in the low clinical risk group and *p* = 0.736 in the high clinical risk group, Figure 4B,C). In the PSM cohort, there were eight recurrence events and no deaths during the median follow-up of 45 months (range, 1–83 months). Likewise, there was no significant difference in RFS according to 21-gene RS status (log-rank test, *p* = 0.850 in the PSM cohort, log-rank test, *p* = 0.583 in the PSM cohort with low clinical risk, and *p* = 0.467 in the PSM cohort with high clinical risk; Figure 4D,F).

## 4. Discussion

Before the era of genomic assays, aggressive chemotherapy was considered for patients at a high clinical risk. However, within node-positive, HR+ breast cancer, several multigene assays revealed that tumors with favorable genomic profiles showed a good prognosis despite a high clinical risk [9,23]. Thus, many investigators wondered assessed whether chemotherapy could be spared in patients with discordant clinical and genomic risk profiles. Concerning this issue, the MINDACT investigators reported no substantial benefit of chemotherapy in patients with clinical high/genomic low risk in terms of distant RFS [12,19]. Moreover, secondary analysis of the TAILOR-X trial showed that the addition of chemotherapy was associated with lower rates of distant recurrence among women with RS of 21–25 and low clinical risk [11].

In this context, it is important to address the influence of 21-gene RS on chemotherapy decision-making according to clinical risk stratification. First, we speculated that 21-gene RS is more helpful in patients with high clinical risk. Although there was no significant difference in chemotherapy rates according to the application of the 21-gene RS in the low clinical risk group, use of the 21-gene RS reduced the chemotherapy rate by approximately 30–40% in the high clinical risk group. Furthermore, the results of the multivariable analysis showed that the application of 21-gene RS was an independent factor for reduced chemotherapy rate in patients at high clinical risk. However, the concerns remain that significant differences in clinicopathologic factors associated with prognosis according to the 21-gene RS in each of the low and high clinical risk groups may influence the chemotherapy rate. Thus, we performed PSM analysis, with similar results: the application of the 21-gene RS reduced the chemotherapy rate by 34% in patients at high clinical risk. Moreover, we also observed no difference in survival outcomes according to the application of 21-gene RS for either clinical risk group. These results suggest that the 21-gene RS could be considered more positively in patients with high clinical risk to reduce chemotherapy rates without increasing the occurrence of relapse.

The trend of reduced chemotherapy rate with the application of the 21-gene RS was more pronounced in N1 patients than N0 patients, regardless of clinical risk. This may occur because N1 patients are strongly recommended to receive chemotherapy unless the 21-gene RS is performed. Accumulating evidence indicates that the 21-gene RS can predict the risk of recurrence and chemotherapy benefit even in N1 patients. N1 patients with RS < 18 who received only endocrine therapy showed comparable 9-year risks of distant recurrence to those with the node-negative disease in the transATAC trial [24]. Similarly, the Surveillance, Epidemiology, and End Results (SEER) and Clalit Health Services registry studies consistently showed favorable 5-year outcomes in N1 patients with RS <18 treated largely without adjuvant chemotherapy [25,26]. Moreover, a retrospective analysis of the SWOG S8814 trial revealed no benefit of chemotherapy in N1 patients with RS < 18 [9]. The RxPONDER trial found that postmenopausal women with N1 and RS ≤ 25 could safely avoid chemotherapy [27]. Together, these data suggest that it is worth applying the 21-gene RS in N1 patients to decrease overtreatment, particularly in postmenopausal women.

In addition, the chemotherapy rates also did not differ according to the use of the 21-gene RS in the low clinical risk group after PSM. This finding could raise another issue regarding the necessity of applying the 21-gene RS assay to determine chemotherapy in patients at low clinical risk. However, it is well established that a high 21-gene RS could predict the clinical benefit from chemotherapy: in the TAILOR-X study, the 21-gene RS identified patients with clinical low risk who benefited from chemotherapy. Further studies integrating clinical and genomic risk profiles with long-term follow are warranted to address the role of the 21-gene RS in patients with low clinical risk.

Our study has a major limitation inherent in retrospective analysis from a single institution with a small proportion of patients who received Oncotype DX assay. In addition, the median follow-up period was relatively short (42 months and 45 months in the PSM cohort), considering that disease-related events occur steadily 5 years after diagnosis in HR+/HER2- breast cancer [28]. Few studies have assessed the survival benefit according to the implementation of the 21-gene RS. To our best knowledge, only one recent study analyzing SEER registry data reported that the application of the 21-gene RS was associated with better breast cancer-specific and overall survivals [29]. However, that study also has limitations, including a median follow-up period of only 36 months and the fact that chemotherapy tended to be underreported in SEER data [30]. Another limitation of the present study was that the chemotherapy decision in our cohort may differ from those made in clinical practice in the future because most patients in our study received treatment before the results of the TAILORx and RxPONDER trials were presented. Lastly, in that era, the guideline for adjuvant treatment was unclear in patients with intermediate RS; thus, the adjuvant treatments may have differed depending on the physicians, which may have affected the clinical outcomes. Therefore, further studies with accurate data collection for adjuvant treatment guided by 21-gene RS and a sufficient follow-up period are needed to clarify whether the application of 21-gene RS influences survival benefits.

## 5. Conclusions

In summary, this is the first study to analyze the usefulness of the 21-gene RS according to clinical risk in the subset of patients with tumor size 1–5 cm, N0-1, HR+/HER2- breast cancer, in terms of chemotherapy rate and survival. The application of the 21-gene RS reduced chemotherapy rates, particularly in patients with high clinical risk. In contrast, it did not alter the chemotherapy rate in patients with low clinical risk in the case-matched cohort. Furthermore, the survival outcomes according to implementation of the 21-gene RS did not differ, even in patients at high clinical risk. These data suggest that the 21-gene RS should be considered positively to reduce overtreatment without adverse prognosis in patients with high clinical risk. For the patients with low clinical risk, further studies with long-term follow-up data are warranted to address the role of the 21-gene RS, which could offer chemotherapy for patients with high genomic risk.

## Figures and Tables

**Table 1:** Baseline characteristics according to clinical risk stratified by 21-gene RS use in the propensity-score matched cohort.

Characteristics	Low Clinical Risk (*n* = 264)				High Clinical Risk (*n* = 200)			
	Patients with 21-Gene RS (*n* = 132)	Patients without 21-Gene RS (*n* = 132)	Total (*n* = 264)	*p*-Value	Patients with 21-Gene RS (*n* = 100)	Patients without 21-Gene RS (*n* = 100)	Total (*n* = 200)	*p*-Value
Age (median)	50	50.5	50	0.405	49	55	51	0.198
Age (range)	25-81	32-86	25-86		27-87	33-75	27-87	
Age (y)				0.902				0.777
≤50	70 (53.0%)	71 (53.8%)	141 (53.4%)		49 (49.0%)	47 (47.0%)	96 (48.0%)	
>50	62 (47.0%)	61 (46.2%)	123 (46.6%)		51 (51.0%)	53 (53.0%)	104 (52.0%)	
Histologic type				>0.999				>0.999
IDC	128 (97.0%)	127 (96.2%)	255 (96.6%)		90 (90.0%)	89 (89.0%)	179 (89.5%)	
ILC	2 (1.5%)	2 (1.5%)	4 (1.5%)		8 (8.0%)	9 (9.0%)	17 (8.5%)	
Others *	2 (1.5%)	3 (2.3%)	5 (1.9%)		2 (2.0%)	2 (2.0%)	4 (2.0%)	
ER, Allred				0.848				0.010
7–8	117 (88.6%)	116 (87.9%)	233 (88.3%)		90 (90.0%)	96 (96.0%)	186 (93.0%)	
0–6	15 (11.4%)	16 (12.1%)	31 (11.7%)		10 (10.0%)	4 (4.0%)	14 (7.0%)	
PR, Allred				0.318				0.671
7–8	73 (55.3%)	81 (61.4%)	154 (58.3%)		52 (52.0%)	55 (55.0%)	107 (53.5%)	
0–6	59 (44.7%)	51 (38.6%)	110 (41.7%)		48 (48.0%)	45 (45.0%)	93 (46.5%)	
Histologic grade				>0.999				0.732
1 or 2	132 (100%)	132 (100%)	264 (100%)		77 (77.0%)	79 (79.0%)	156 (78.0%)	
3	0	0	0		23 (23.0%)	21 (21.0%)	44 (22.0%)	
LVI ^†^				>0.999				0.884
Yes	12 (9.1%)	12 (9.1%)	24 (9.1%)		37 (37.0%)	38 (38.0%)	75 (37.5%)	
No	120 (90.9%)	120 (90.9%)	240 (90.9%)		63 (63.0%)	62 (62.0%)	125 (62.5%)	
Ki-67				0.295				0.768
≥14	32 (24.2%)	25 (18.9%)	57 (21.6%)		65 (65.0%)	63 (63.0%)	128 (64.0%)	
<14	100 (75.8%)	107 (81.1%)	207 (78.4%)		35 (35.0%)	37 (37.0%)	72 (36.0%)	
T stage				>0.999				0.556
1	129 (97.7%)	128 (97.0%)	257 (97.3%)		38 (38.0%)	34 (34.0%)	72 (36.0%)	
2	3 (2.3%)	4 (3.0%)	7 (2.7%)		62 (62.0%)	66 (66.0%)	128 (64.0%)	
*n* stage				>0.999				0.888
0	122 (92.4%)	122 (92.4%)	244 (92.4%)		52 (52.0%)	51 (51.0%)	103 (51.5%)	
1	10 (7.6%)	10 (7.6%)	20 (7.6%)		48 (48.0%)	49 (49.0%)	97 (48.5%)	

* Others (*n* = 9) included mucinous (*n* = 5), tubular (*n* = 2), and papillary (*n* = 2) breast cancers. **^†^** Missing values. Abbreviations: RS, recurrence score; IDC, invasive ductal carcinoma; ILC, invasive lobular carcinoma; ER, estrogen receptor; PR, progesterone receptor; LVI, lymphovascular invasion.

**Table 2:** Adjusted odds ratios (ORs) and 95% confidential intervals (CIs) of 21-gene RS for the implementation of chemotherapy in patients with high clinical risk among the entire cohort.

	Multivariable Analysis					
21-Gene RS	All Patients		Node-Negative		Node-Positive	
	Odds Ratio (95% CIs)	*p*-Value *	Odds Ratio (95% CIs)	*p*-Value ^†^	Odds Ratio (95% CIs)	*p*-Value ^‡^
No	Ref.		Ref.		Ref.	
Yes	0.196 (0.115–0.334)	<0.001	0.357 (0.176–0.724)	0.004	0.085 (0.036–0.200)	<0.001

Abbreviations: ER, estrogen receptor; PR, progesterone receptor; LVI, lymphovascular invasion; RS, recurrence score. * Covariates for multivariable models were histologic type, ER, PR, histologic grade, LVI, Ki-67, and *n* stage. **^†^** Covariates for multivariable models were histologic type, ER, PR, histologic grade, Ki-67, and T stage. **^‡^** Covariates for multivariable models were ER, PR, Ki-67, and T stage.

## Data Availability

The data that support the findings of this study, contain clinical outcomes for which IRB requires approval prior to analysis. Therefore, the data are not publicly available. The data will be made available to authorized researchers who have obtained institutional review board (IRB) approval from their own institution and from Gangnam Severance Hospital, Yonsei University, Seoul, Republic of Korea IRB. For data access requests, please contact the corresponding author, Joon Jeong, email address: gsjjoon@yuhs.ac.

## Supplementary Materials

The following are available online at https://www.mdpi.com/article/10.3390/cancers13164003/s1, Figure S1: Distributions of the 21-gene Recurrence Score (RS) according to the clinical risks, Table S1: Baseline characteristics according to clinical risk stratified by use of the 21-gene Recurrence Score (RS) assay.
