# Supplementary material for: Application of the 21-Gene Recurrence Score in Patients with Early HR-Positive/HER2-Negative Breast Cancer: Chemotherapy and Survival Rate According to Clinical Risk
Source: Cancers (Basel). 2021 Aug 9;13(16):4003. doi: 10.3390/cancers13164003 (PMC8394098; doi:10.3390/cancers13164003)
Supplement: Supplementary file 1 [file cancers-13-04003-s001.zip › cancers-1267930-supplementary.pdf]

Supplementary Materials

Table 1. Baseline characteristics according to clinical risk stratified by use of the 21-gene Recurrence Score (RS) assay.

	All patients				Low clinical risk				High clinical risk			
Characteristics	Clinical risk low (n=536)	Clinical risk high (n=342)	Total (n=878)	*p*-value	Patients with 21-gene RS (n=174)	Patients without 21-gene RS (n=362)	Total (N=536)	*p*-value	Patients with 21-gene RS (n=144)	Patients without 21-gene RS (n=198)	Total (N=342)	*p*-value
Age (y)				0.683				0.992				0.104
≤50	274 (51.1%)	170 (49.7%)	444 (50.6%)		185 (51.1%)	89 (51.1%)	274 (51.1%)		91 (46.0%)	79 (54.9%)	170 (49.7%)	
>50	262 (48.9%)	172 (50.3%)	434 (49.4%)		177 (48.9%)	85 (48.9%)	262 (48.9%)		107 (54.0%)	65 (45.1%)	172 (50.3%)	
Histologic type				0.022				0.002				0.027
IDC	453 (84.5%)	289 (84.5%)	742 (84.5%)		159 (91.4%)	294 (81.2%)	453 (84.5%)		126 (84.5%)	163 (82.3%)	289 (84.5%)	
ILC	30 (5.6%)	32 (9.4%)	62 (7.1%)		9 (5.2%)	21 (5.8%)	30 (5.6%)		15 (10.4%)	17 (8.6%)	32 (9.4%)	
Others*	53 (9.9%)	21 (6.1%)	74 (8.4%)		6 (11.3%)	47 (13.0%)	53 (13.0%)		3 (2.1%)	18 (9.16%)	21 (6.1%)	
ER, Allred				0.289				0.118				0.044
7-8	481 (89.7%)	299 (87.4%)	780 (88.8%)		151 (86.8%)	330 (91.2%)	481 (89.7%)		132 (91.7%)	167 (84.3%)	299 (87.4%)	
0-6	55 (10.3%)	43 (12.6%)	98 (11.2%)		23 (13.2%)	32 (8.8%)	55 (10.3%)		12 (8.3%)	31 (15.7%)	43 (12.6%)	
PR, Allred				0.006				<0.001				0.058
7-8	302 (56.3%)	160 (46.8%)	462 (52.6%)		65 (37.4%)	237 (65.5%)	302 (56.3%)		76 (52.8%)	84 (42.4%)	160 (46.8%)	
0-6	234 (43.7%)	182 (53.2%)	416 (47.4%)		109 (62.6%)	125 (34.5%)	234 (43.7%)		68 (47.2%)	114 (57.6%)	182 (53.2%)	
Histologic grade				<0.001				0.452				0.517
1 or 2	532 (99.3%)	260 (76.0%)	792 (90.2%)		172 (98.9%)	360 (99.4%)	532 (99.3%)		112 (77.8%)	148 (74.7%)	260 (76.0%)	
3	4 (0.7%)	82 (24.0%)	86 (9.8%)		2 (1.1%)	2 (0.6%)	4 (0.7%)		32 (22.2%)	50 (25.3%)	82 (24.0%)	
LVI†				<0.001				0.820				0.011
Yes	55 (10.3%)	126 (37.0%)	181 (20.7%)		155 (90.1%)	323 (89.5%)	478 (89.7%)		42 (29.2%)	84 (42.6%)	126 (37.0%)	
No	478 (89.7%)	215 (63.0%)	693 (79.3%)		17 (9.9%)	38 (10.5%)	55 (10.3%)		102 (70.8%)	113 (57.4%)	215 (63.0%)	
Ki-67				<0.001				<0.001				0.066
≥14	95 (17.7%)	131 (38.3%)	226 (25.7%)		53 (30.5%)	42 (11.6%)	95 (17.7%)		47 (32.6%)	84 (42.4%)	131 (38.3%)	
<14	441 (82.3%)	211 (61.7%)	652 (74.3%)		121 (69.5%)	320 (88.4%)	441 (82.3%)		97 (67.4%)	114 (57.6%)	211 (61.7%)	
T stage				0.058				0.960				0.951
1	509 (95.0%)	113 (33.0%)	622 (70.8%)		166 (95.4%)	345 (95.3%)	511 (95.3%)		47 (32.6%)	64 (32.3%)	111 (32.5%)	
2	27 (5.0%)	229 (67.0%)	256 (29.2%)		8 (4.6%)	17 (4.7%)	25 (4.7%)		97 (67.4%)	134 (67.7%)	231 (67.5%)	
N stage				<0.001				0.246				0.002

0	505 (94.2%)	182 (53.2%)	687 (78.2%)		161 (92.5%)	344 (95.0%)	505 (94.2%)		91 (63.2%)	91 (46.0%)	182 (53.2%)	
1	31 (5.8%)	160 (46.8%)	191 (21.8%)		13 (7.5%)	18 (5.0%)	31 (5.8%)		53 (36.8%)	107 (54.0%)	160 (46.8%)	
Chemotherapy				<0.001				0.059				0.005
Yes	71 (13.2%)	180 (52.6%)	251 (28.6%)		30 (17.2%)	41 (11.3%)	71 (13.2%)		43 (29.9%)	137 (69.2%)	180 (52.6%)	
No	465 (86.8%)	162 (47.4%)	627 (71.4%)		144 (82.8%)	321 (88.7%)	465 (86.8%)		101 (70.1%)	61 (30.8%)	162 (47.4%)	
21-gene RS				0.004				-				-
Yes	174 (32.5%)	144 (42.1%)	318 (36.2%)		-	-	-		-	-	-	
No	362 (67.5%)	198 (57.9%)	560 (63.8%)		-	-	-		-	-	-	

*Others (n=74) included mucinous (n=38), tubular (n=17), papillary (n=18), and metaplastic (n=1) breast cancers.

†Missing values.

Abbreviations: RS=recurrence score, IDC=invasive ductal carcinoma, ILC=invasive lobular carcinoma, ER=estrogen receptor, PR=progesterone receptor, LVI=lymphovascular invasion.

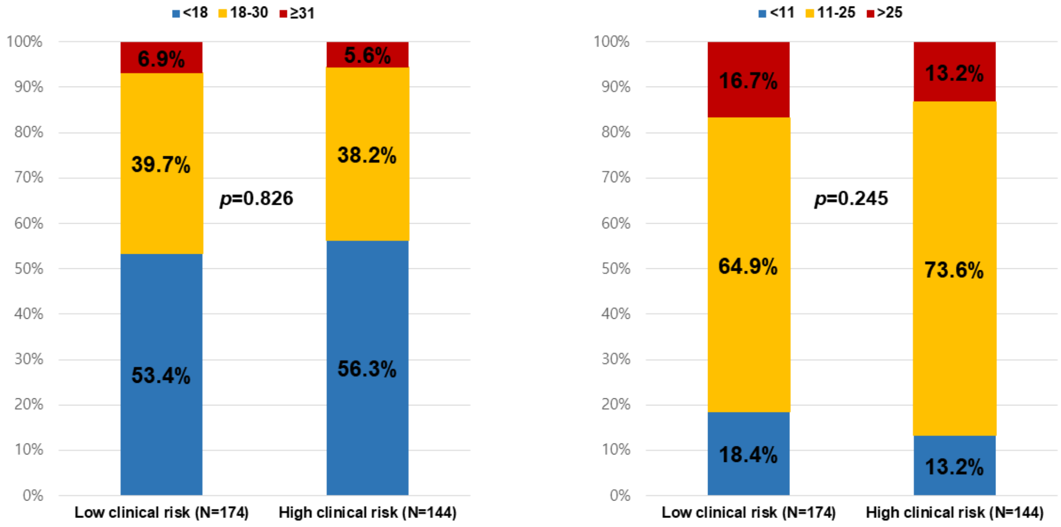

Figure 1. Distributions of the 21-gene Recurrence Score (RS) according to the clinical risks. (A) Cut-off scores for low, intermediate, and high RS of <18, 18–30, and ≥31, respectively. (B) Cut-off scores for low, intermediate, and high RS of <11, 11–25, and high >25, respectively. *Chi-square test, significance level 0.05.*
